# The Metabolic Burden of Methyl Donor Deficiency with Focus on the Betaine Homocysteine Methyltransferase Pathway

**DOI:** 10.3390/nu5093481

**Published:** 2013-09-09

**Authors:** Rima Obeid

**Affiliations:** Department of Clinical Chemistry, University Hospital of the Saarland, D-66424, Homburg, Germany; E-Mail: rima.obeid@uniklinikum-saarland.de; Tel.: +49-0-6841-1630-711; Fax: +49-0-6841-1630-703

**Keywords:** folate, betaine, choline, methyl, energy, lipids

## Abstract

Methyl groups are important for numerous cellular functions such as DNA methylation, phosphatidylcholine synthesis, and protein synthesis. The methyl group can directly be delivered by dietary methyl donors, including methionine, folate, betaine, and choline. The liver and the muscles appear to be the major organs for methyl group metabolism. Choline can be synthesized from phosphatidylcholine via the cytidine-diphosphate (CDP) pathway. Low dietary choline loweres methionine formation and causes a marked increase in *S*-adenosylmethionine utilization in the liver. The link between choline, betaine, and energy metabolism in humans indicates novel functions for these nutrients. This function appears to goes beyond the role of the nutrients in gene methylation and epigenetic control. Studies that simulated methyl-deficient diets reported disturbances in energy metabolism and protein synthesis in the liver, fatty liver, or muscle disorders. Changes in plasma concentrations of total homocysteine (tHcy) reflect one aspect of the metabolic consequences of methyl group deficiency or nutrient supplementations. Folic acid supplementation spares betaine as a methyl donor. Betaine is a significant determinant of plasma tHcy, particularly in case of folate deficiency, methionine load, or alcohol consumption. Betaine supplementation has a lowering effect on post-methionine load tHcy. Hypomethylation and tHcy elevation can be attenuated when choline or betaine is available.

## 1. Introduction

Hypomethylation has a wide spectrum of effects that include genetic, epigenetic, and metabolic alterations. Dietary methyl donors or endogenously produced methyl groups are promising for disease prevention and risk modification.

## 2. Need for Methyl Groups

*S*-adenosylmethionine (SAM) is the major methyl donor in the cell. It is involved in numerous cellular reactions, including DNA methylation and synthesis of phosphatidylcholine, and in reactions involving neurotransmitters, creatine, carnitine, and antioxidants (such as glutathione and taurine). Methionine, betaine, choline, and 5-methyltetrahydrofolate (5-MTHF) are important dietary sources of labile methyl groups in mammalian cells.

**Figure 1:**
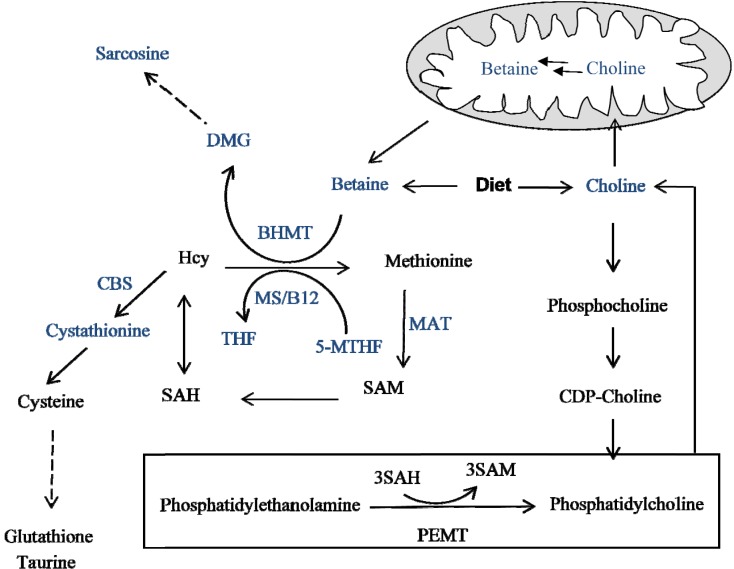
Methylation cycle. BHMT: betaine homocysteine methyltransferase, CBS: cystathionine beta synthase, DMG: dimethylglycine, Hcy: homocysteine, MAT: l-methionine *S*-adenosyltransferase, MS: methionine synthase, PEMT: phosphatidylethanolamine methyltransferase, 5-MTHF: 5-methyltetrahydrofolate, SAH: *S*-adenosylhomocysteine, SAM: *S*-adenosylmethionine, THF: tetrahydrofolate.

SAM is produced from methionine by l-methionine *S*-adenosyltransferase (MAT) (Figure 1). Methionine is supplied either by the diet or is generated from homocysteine via methionine synthase or via betaine homocysteine methyltransferase (BHMT). The BHMT pathway is particularly active in the liver and the kidney, which are the main organs that store large amounts of betaine. During methyl group donation, SAM is converted into *S*-adenosylhomocysteine (SAH), a potent competitive inhibitor of many methyltransferases [1].

The transmethylation cycle is strongly regulated by feedback mechanisms. For example, SAM activates cystathionine beta synthase to enhance the conversion of homocysteine into cysteine, the precursor of glutathione and taurine. In contrast, SAM inhibits BHMT, thus reducing the utilization of betaine as a methyl donor. Moreover, SAM inhibits methylenetetrahydrofolate reductase (MTHFR), thereby reducing the availability of 5-MTHF as a methyl donor. The product of betaine demethylation, dimethylglycine (DMG), inhibits BHMT and controls methyl group transfer. Methionine load activates the transsulfuration pathway, whereas the remethylation pathway via methionine synthase is active under fasting conditions.

The process of methylogenesis depends on several nutrients such as folate, methionine, vitamins B12, B6, and B2, betaine, and choline. The methylation potential (SAM/SAH ratio) is rather resistant to fluctuations in independent nutrients [2]. A study on rural African women showed that seasonal variations were ameliorated by the intake of the dietary methyl donors such as folate, betaine, and vitamins B6 and B12 [2]. Moreover, there appeared to be a seasonal switch between the folate and betaine pathways as sources of methyl groups in this study on African women [2].

## 3. Methyl Donor: Folate

Folate (vitamin B9) is a member of the B-vitamin group that is required for crucial cell processes. Green leafy plants are rich sources of folate in human diet. The recommended daily intake of folate in adults is 400 µg/day. Formyltetrahydrofolate and methenyltetrahydrofolate are utilized in the synthesis of purine and pyrimidine, respectively. Moreover, 5-MTHF plays a key role as a methyl donor. Three enzymes are required to produce methyl groups via the folate cycle: serine hydroxymethyl transferase (SHMT), MTHFR, and methionine synthase. Methionine synthase, a vitamin B12-dependent enzyme, facilitates the last step of methyl group transfer from 5-MTHF to homocysteine to form methionine. Folate deficiency causes anemia and depression, and increases the risk of pregnancy complications and birth defects [3,4]. A recent meta-analysis suggested an association between the C677T polymorphism in the MTHFR gene and neural tube defects, independent of folate status [5]. Inactivation of 10-formyltetrahydrofolate synthetase lowered purine synthesis and slowed cell proliferation [6]. Maternal Mthfd1 disruption impairs fetal growth but was not associated with birth defects [7]. Approximately 30%–70% of neural tube defects can be prevented by taking folic acid supplementations before conception, though the mechanisms of the preventive role of folate have not been thoroughly studied. Folate deficiency causes several metabolic changes in the cell, including hyperhomocysteinemia, low SAM levels, and DNA hypomethylation [8,9].

## 4. Methyl Donors: Betaine and Choline

Betaine is the methyl donor in the BHMT pathway and is a methylamine as it contains three chemically reactive methyl groups linked to a nitrogen atom. Wheat bran, wheat germ, spinach, and beets are rich sources of betaine in the human diet. Betaine intake varies between populations (range: 100–400 mg/day) [10,11]. Much lower intakes (mean, 33 mg/day) have been reported in one recent study on rural African women [2], suggesting that the regulation of the methylation cycle may differ according to lifestyle and environmental factors. Moreover, differences in intake data between populations may be related to under- or over-estimation of the food contents of betaine and choline.

In addition to dietary sources, betaine can be produced through the irreversible oxidation of choline via choline dehydrogenase and betaine aldehyde dehydrogenase. Betaine is converted into DMG as it donates a methyl group to homocysteine. This pathway is mediated by BHMT, an enzyme induced by betaine [12] and inhibited by DMG or SAM [13]. A small amount of DMG is eliminated in the urine, and most DMG is converted via DMG-dehydrogenase into sarcosine and glycine. DMG-dehydrogenase is a mitochondrial flavoprotein and folate-binding protein. It transfers one-carbon unit to tetrahydrofolate to produce 5,10-methylenetetrahydrofolate. The non-essential amino acid glycine enters the folate cycle to produce 5,10-methylenetetrahydrofolate or is used to synthesize bile salts, glutathione, and proteins such as collagen. Alternatively, glycine is used for creatine synthesis via guanidinoacetate in a SAM-dependent pathway [14]. The flow of methyl groups via the BHMT pathway is not dependent on folate or vitamin B12, but there are several cross-talk mechanisms between the BHMT and the folate pathways. Choline has been shown to indirectly support homocysteine methylation [15]. One study of postmenopausal women showed that choline supplementation increased plasma betaine levels and slightly lowered plasma levels of total homocysteine (tHcy) after six weeks (median change, −0.9 *vs.* +0.6 μmol/L in the placebo group: *p* = 0.058) and 12 weeks [15]. Sex- and age-related differences in choline requirements and blood levels have been reported. Women have lower plasma concentrations of betaine and choline than men, as well as younger subjects, showed lower betaine concentrations than older subjects [16].

Red meat, poultry, milk, egg, and fish are rich sources of choline [10]. The estimated recommended intake for choline for adults is 550 mg/day for men, 425 mg/day for women, 450 mg/day for pregnant women, and 550 mg/day for lactating women [17]. The upper tolerable limit for adults is 3.5 g/day. In addition to the diet, a main source of choline is *de novo* synthesis from phosphatidylcholine via the CDP-pathway. Phosphatidylcholine can be formed either from choline or from phosphatidylethanolamine via the phosphatidylethanolamine methyltransferase (PEMT) pathway. Choline generation via PEMT consumes a significant amount of SAM (3 mole SAM to produce 1 mole choline) and produces homocysteine. Accordingly, PEMT knockout mice show lower plasma concentrations of tHcy [18]. In contrast, mice deficient in liver CTP:phosphocholine cytidylyltransferase have high tHcy and low phosphatidylcholine because of low production from choline [19]. SAM concentrations are maintained by stimulating the BHMT pathway [19].

Available evidence suggests that folate deficiency can be partly attenuated when choline is available and vice versa. For example, choline and phosphatidylcholine were depleted in the livers of rats fed a folate-deficient diet [20]. In contrast, consumption of a choline-deficient diet decreased hepatic folate stores [21]. A choline-deficient diet lowered methionine formation in animal livers by 20%–25% [22], probably because less choline was available for conversion into betaine. However, the effects of choline deficiency on reducing liver SAM (by 60%) and increasing liver SAH (by 50%) were impressive [22]. Therefore, the effect of choline deficiency on lowering SAM is probably not solely mediated by lowering methionine. A choline-deficient diet may increase SAM utilization in the liver, to convert phosphatidylethanolamine into phosphatidylcholine via PEMT.

The liver and the muscles are the major sites of choline metabolism. Choline deficiency caused fatty liver and muscle damage in humans and increased hepatic carcinogenesis in rodents exposed to alcohol [23].

## 5. Metabolic Burden of a Methyl-Deficient Diet

Studies simulating methyl-deficient diets have reported disorders in protein synthesis in the liver and fatty liver as well as muscle disturbances [24,25,26]. Betaine [27,28,29,30], choline, or folate [31] were able to reverse alcohol-related or non-alcohol related liver disturbances, probably via epigenetic regulation [32] or lipid-related mechanisms. The quantitative participation of each of the methyl donors in the daily net flow of methyl groups has not been thoroughly examined [33,34]. Large fluctuations in plasma concentrations of folate and betaine were ameliorated and not translated to large fluctuations in the plasma SAM/SAH ratio [2]. However, the plasma SAM/SAH ratio did not show significant changes, and it is currently not known whether this ratio reflects the methylation potential in all organs.

Betaine is stored in the cell and hence represents a ready-to-use methyl group source. Animal studies have shown that plasma betaine is a poor predictor of tissue betaine content [35]. DMG concentrations in plasma may be a good indicator of betaine utilized as a methyl donor [2]. For example, plasma betaine was increased in only 36% of folate-deficient patients and in 12% of folate-deficient patients who had severe alcoholic liver disease [36]. Plasma DMG was increased in 74% of the folate-deficient patients [36], suggesting that plasma DMG rather than betaine may be a good marker for betaine utilization as a methyl donor [36].

Despite the questionable importance of low and high plasma betaine [37], plasma betaine is a significant determinant of plasma tHcy concentration. A recent study in a large sample of pregnant women showed that women with low plasma folate at 24–27 gestational weeks (<11.4 nmol/L) had lower plasma betaine and higher DMG and tHcy until the end of pregnancy compared to women with plasma folate above 11.4 nmol/L [38]. Plasma betaine is a negative predictor of increased plasma tHcy after methionine load in subjects with low folate [39]. Moreover, a low methionine diet slightly enhanced the BHMT pathway rather than the transsulfuration pathway [40]. Similarly, in cases of folate deficiency, the BHMT pathway appears to be a feasible method for re-methylating homocysteine after a methionine load [39,41]. In contrast, folate supplementation causes a dose-dependent increase in plasma betaine [42], suggesting that betaine is being utilized to a lower extent. In non-supplemented individuals, the concentrations of folate and betaine showed no correlation, and plasma folate was not a significant predictor of plasma betaine [42]. Another study examining healthy individuals (mean age, 36 years) who were not receiving supplements revealed a direct relationship between plasma betaine and choline and that of serum folate, but no relationship with plasma tHcy [43]. Plasma SAM concentrations were positively correlated with plasma levels of choline and DMG, but not with that of betaine [43].

Changes in tHcy concentrations reflect only one side of the metabolic burden of methyl group deficiency or nutrient supplementation. Folate [16], betaine [16,33,44], and choline [45] are significant determinants of the fasting plasma concentrations of tHcy. Betaine and choline intake were inversely related to fasting and post-methionine load tHcy concentrations [46]. This inverse correlation was no longer present in populations ingesting foods fortified with folic acid [46]. As folic acid lowers plasma tHcy, betaine is probably more effective as a methyl donor and as a tHcy-lowering nutrient in populations not on fortification programs compared to those on such programs [46]. One study showed that plasma concentrations of tHcy and betaine are inversely correlated in healthy men and women [42]. Supplementing folic acid 400–800 µg/day for 12 weeks caused a 15% increase in plasma betaine levels, but the inverse correlation between tHcy and betaine remained significant after supplementation [42]. In general betaine seems to be less involved in tHcy methylation in people who received folic acid supplementation than in those who did not receive folate supplementation. In agreement with these results, in subjects with low serum folate, concentrations of fasting or post-methionine load tHcy and that of betaine showed a pronounced inverse association [16]. Plasma folate is a more important determinant of plasma tHcy than plasma betaine [16]. Furthermore, acute supplementation of a small dose of 550 mg betaine slightly lowered circulating tHcy and increased DMG, with minimal levels of betaine appearing in the urine, suggesting that betaine was metabolized and partly stored in tissues [42]. Only a small amount is excreted in the urine, even when supplementation is continued [42]. Acute supplementation of betaine (550 mg) had a stronger lowering effect on post-methionine load tHcy concentrations in one study on healthy men [42]. In a further study that included patients with renal failure, a large dose of betaine (4 g/day) for three months caused no additional lowering of tHcy when given with 5 mg folic acid and 50 mg B6 compared to 5 mg folic acid plus 50 mg B6 without betaine [41]. However, betaine increased plasma and urine betaine and DMG in this study [41], suggesting that at least some of the betaine was metabolized to DMG.

Phosphatidylcholine supplementation for two weeks in healthy men from a population without mandatory fortification with folic acid lowered mean plasma tHcy by 18% [−3.0 (−3.9, −2.1) µmol/L] and mean post-methionine load tHcy by 29% [−9.2 (−11.3, −7.2) µmol/L] compared to the placebo [47]. The intake of glycerophosphocholine (mainly obtained from milk) was negatively related to plasma tHcy in women with low intakes of folate (<400 µg/day) (mean tHcy 12.0 *vs.* 10.2 µmol/L: −15%) [11]. Alcohol consumption (≥15 g/day) was also a strong predictor of the inverse association between choline and tHcy in women with low folate intake [11]. Alcohol is known to inhibit methionine synthase activity in the liver, thus increasing plasma tHcy, lowering liver SAM, and causing fatty liver [48]. Therefore, the BHMT pathway becomes more important as a source of SAM and determinant of tHcy in alcoholism [48].

The effect of folate in preventing tHcy elevation was recently tested in BHMT deleted mice (Bhmt^−/−^) [49]. Bhmt^−/−^ mice showed approximately 10-fold higher plasma tHcy, heavier liver (fatty liver), lower liver SAM, and higher liver SAH than Bhmt^+/+^ mice [49]. tHcy elevation was not corrected after folic acid supplementation. However, folic acid supplemented Bhmt^−/−^ mice were able to produce more hepatic SAM compared to Bhmt^−/−^ mice fed a folate-deficient diet or those on a control diet [49]. The results suggest that folate enhanced SAM production via the methionine synthase pathway, but was not able to remove tHcy in this mouse model. The BHMT pathway appears to substantially participate in tHcy metabolism in mice [50]. At least in this mouse model, folic acid supplementation did not counterbalance the metabolic burden caused by disrupting the BHMT enzyme.

Taken together, betaine and choline support the role of folate in lowering fasting tHcy. The effect of betaine on lowering post-methionine load tHcy is stronger than its effect on fasting tHcy [41]. The tHcy-lowering effect of betaine is higher in individuals not receiving folic acid. The effect of betaine on plasma tHcy appears to be more dependent on plasma folate than on plasma betaine [51]. In folate-deficient patients, plasma betaine and methionine concentrations were maintained and DMG was increased, suggesting that betaine acted as a methyl donor to produce methionine and DMG [51]. Plasma betaine does not reflect tissue betaine, but may reflect transient changes in the labile methyl group in case of higher requirements (pregnancy, liver damage), after methionine-load, or after folic acid supplementation.

## 6. Metabolic Burden: Osmotic Stress and Energy Metabolism

### 6.1. Osmotic Stress

Betaine is a major osmolyte in the cell [52]; it regulates the cell volume and stabilizes proteins. It is stored at high amounts in the liver and the kidney. Betaine levels in skeletal muscles are similar to those in the plasma and brain [35]. Betaine prevents osmolytic stress when added to farmed fish upon transfer from low to high salinity. Moreover, in cells exposed to hyperosmotic conditions, choline uptake is enhanced in mitochondria in order to resist volume reduction and osmotic water loss. Choline is converted into betaine under these conditions [53].

### 6.2. Energy and Lipid Metabolism

Hypomethylation is involved in energy metabolism disorders. Much on betaine effects can be learned from animal studies. Betaine has been an important component of animal nutrition for over 50 years. A choline-rich diet is an additional source of betaine since choline is oxidized to betaine by the mitochondrial enzyme choline dehydrogenase [54] and betaine aldehyde dehydrogenase, which is expressed in the cytosol and mitochondria.

Betaine was shown to be related to protein (muscular tissue) and fat metabolism [55]. Moreover, betaine has been discussed in animal nutrition for its role in saving methionine and choline [56,57]. The methionine-sparing effect of betaine makes methionine more available for protein synthesis, and the choline-sparing effect makes choline more available for lipid metabolism. The role of betaine in energy metabolism appears to be effective at low total energy intake. Betaine is useful as a partitioning agent under low amino acid and energy intake situations in animals [56,57]; thus, it has the potential to solve public health problems related to excess fat in meat products.

Betaine is considered to be a lipotropic compound that can influence lipid metabolism in animals [58]. It improves energy metabolism probably by supporting the synthesis of carnitine, which is necessary for the transport of long-chain fatty acids to the mitochondria where they are oxidized [58]. Löest *et al.* found that betaine may decrease the demand for choline methyl groups, thus increasing choline availability for lipid metabolism [59]. This was supported by the findings of Yao and Vance [60] showing that betaine can correct very low density lipoprotein (VLDL) secretion from hepatocytes grown in a choline deficient medium.

Human studies have demonstrated a relationship between low betaine and BMI. In one large population-based study, BMI was inversely related to betaine and directly related to choline [61]. The same was found for body fat and triglycerides [61]. In a further study, plasma betaine was lower in men with BMI ≥ 25 kg/m^2^ compared with men with BMI < 25 kg/m^2^ (median 37.3 *vs.* 40.1: *p* = 0.049), but plasma choline did not differ significantly [62]. In obese men, plasma betaine was inversely related to blood lipids and liver enzymes [62], suggesting that betaine (or choline) can ameliorate fatty liver and the components of metabolic syndrome. The distribution of choline between the methylation and phospholipid pathways appears to play a key role in energy metabolism since the association of alanine aminotransferase (ALT is a liver enzyme) and low-density lipoprotein (LDL)-cholesterol showed a negative association with the betaine/choline ratio only in individuals with a polymorphism in PEMT (PEMT5465 GG) [62]. A genotype-phenotype-specific relationship between choline metabolites and metabolic stress has been suggested [62], but needs to be confirmed.

A very high-methionine/low-folate diet caused hyperhomocysteinemia and accumulation of cholesterol and triacylglycerol in mouse liver but not in the plasma [63]. Plasma VLDL was increased, suggesting that the excretion of VLDL from the liver was not impaired [63]. Activation of both the unfolded protein response and sterol regulatory element-binding proteins has been suggested. In cases of higher plasma folate, choline is probably spared and utilized for phospholipid synthesis. This can explain results on the inverse association between plasma folate and plasma lipoproteins (LDL-cholesterol) [64], or the increased high-density lipoprotein (HDL)-cholesterol levels after folic acid supplementation [65].

A moderate high-fat diet (20% of total calories from fat) in mice caused a higher body weight, blood glucose, triglycerides, and insulin compared with a low-fat diet (9% of total calories from fat) [28]. Betaine supplementation was able to prevent and reverse fatty liver, lower serum ALT, and improve insulin resistance in mice fed a high-fat diet for up to eight months [28]. Betaine was also able to correct insulin signaling by activating insulin receptor substrate 1 (IRS1) by increasing tyrosine phosphorylation and other signaling mechanisms that regulate gluconeogenesis and glycogen synthesis [28].

The role of betaine and choline in energy metabolism appears to be beyond the effect on gene methylation and epigenetic control since folate is not known to show the same effect on energy metabolism, but rather on epigenetic events.

## 7. Methyl Donor and Physiological and Disease Conditions

Physiological changes in plasma folate and tHcy during pregnancy have been reported previously. Pregnancy-related changes in plasma betaine and choline have also been reported [38,66]. From gestational week eight onward, choline increased during pregnancy while betaine decreased. From 20 gestational weeks onward, betaine was a stronger predictor of plasma tHcy than folate [66]. Increased plasma choline during pregnancy [38] is probably related to enhanced endogenous synthesis, since PEMT enzyme is induced by estrogen. Decreased betaine during pregnancy is associated with increased DMG suggesting that a significant amount of methyl groups are afforded by the BHMT pathway at late gestation [38]. The role of the BHMT pathway in delivering methyl groups may be even more significant in pregnant women with low folate [38]. Folate [67] and choline have been related to cognitive development and brain function. In a recent report from the Hordaland Health Study on 2190 participants, better cognitive function was associated with higher choline and B12 or higher betaine and B12 [68].

Folate or choline deficiency has been related to several adverse health effects in humans. The association between folate deficiency and cancer is also well-documented [69]. The most important diseases associated with folate deficiency are neural tube defects. Choline deficiency or defects in choline metabolism have been also linked to fetal brain development and birth defects [70,71]. Defects in choline utilization cause early embryonic lethality and have severe effects on embryo survival or development [72]. Furthermore, severe heart defects were observed when a choline deficient diet (1/8 of the recommended daily intake) was administered to mice, six weeks before conception [73]. Folate and choline can prevent disorders in brain development and function, at least in part by supplementing methyl groups.

Both high and low plasma concentrations of betaine are linked to diseases [37]. Urinary betaine excretion is increased in approximately 20% of patients with type 2 diabetes [74]. Low levels of plasma betaine are associated with cardiovascular risk factors and probably cardiovascular risk [44].

Several mechanisms may explain the role of dietary methyl donors in human diseases. For example, hypo- or hyper methylation of DNA and disruption of DNA repair were reported in cancer patients and were related to methyl donors [75,76]. Disturbed methylation of key proteins in the brain plays a central role in the neurodegenerative process and enhances the pathology of amyloid beta and the tau protein [77]. Other mechanisms may be related to the toxic effects of hyperhomocysteinemia or oxidative stress. Osmotic stress caused by low intracellular betaine can affect protein stability and signaling pathways in the cell [28]. Disruption of the methylation pathway in the liver (by alcohol or choline deficiency) causes accumulation of lipids and fatty liver. Finally, mechanisms not related to methylation may be involved. For example, choline is used for synthesizing the neurotransmitter acetylcholine and the important membrane lipid, phosphatidylcholine.

## 8. Conclusions

Metabolism of folate, betaine, choline, and methionine are interrelated, and deficiency of one nutrient can be partly compensated for. Deficiency of dietary methyl donors can cause metabolic and functional disturbances. Metabolic changes in the cell are reflected by elevated tHcy levels, disturbed energy and lipid metabolism, and dysregulation of DNA methylation and protein synthesis. Folate supplements save betaine as a methyl donor, and betaine supplementation appears to have a lowering effect on post-methionine loading of tHcy. The association between fasting plasma tHcy and plasma betaine or choline appears to be equivocal; however, betaine or choline supplementation can lower plasma tHcy.
